# Investigation of the Anti-*Leishmania* (Leishmania) *infantum* Activity of Some Natural Sesquiterpene Lactones

**DOI:** 10.3390/molecules22050685

**Published:** 2017-04-25

**Authors:** Imke F. Wulsten, Thais A. Costa-Silva, Juliana T. Mesquita, Marta L. Lima, Mariana K. Galuppo, Noemi N. Taniwaki, Samanta E. T. Borborema, Fernando B. Da Costa, Thomas J. Schmidt, Andre G. Tempone

**Affiliations:** 1 Department of Biology, Chemistry, Pharmacy, Freie Universität Berlin, 14195 Berlin, Germany; imkefwulsten@zedat.fu-berlin.de; 2 Centre for Parasitology and Mycology, Instituto Adolfo Lutz (IAL), Av. Dr. Arnaldo, 351, CEP 01246-900 São Paulo, SP, Brazil; tha_isbio@yahoo.com.br (T.A.C.-S.); jt_mesquita@hotmail.com (J.T.M.); martallima@usp.br (M.L.L.); marianakg7@gmail.com (M.K.G.); ntaniwak@hotmail.com (N.N.T.); samanta@usp.br (S.E.T.B.); 3 Instituto de Medicina Tropical, Universidade de São Paulo, Av. Dr. Enéas de Carvalho Aguiar, 470, CEP 05403-000 São Paulo, SP, Brazil; 4 AsterBioChem Research Team, Laboratory of Pharmacognosy, School of Pharmaceutical Sciences of Ribeirão Preto, USP, Av. do Café s/n, 14040-903 Ribeirão Preto, SP, Brazil; febcosta@fcfrp.usp.br; 5 Institute of Pharmaceutical Biology and Phytochemistry (IPBP), University of Münster, PharmaCampus, Corrensstraße 48, 48149 Münster, Germany

**Keywords:** sesquiterpene lactones, pseudoguaianolides, helenalin acetate, mexicanin I, *Leishmania* (L.) *infantum*, drugs, nitric oxide

## Abstract

Leishmaniases are neglected infectious diseases caused by parasites of the ‘protozoan’ genus *Leishmania*. Depending on the parasite species, different clinical forms are known as cutaneous, muco-cutaneous, and the visceral leishmaniasis (VL). VL is particularly fatal and the therapy presents limitations. In the search for new anti-leishmanial hit compounds, seven natural sesquiterpene lactones were evaluated against promastigotes and intracellular amastigotes of *Leishmania* (Leishmania) *infantum*, a pathogen causing VL. The pseudoguaianolides mexicanin I and helenalin acetate demonstrated the highest selectivity and potency against intracellular amastigotes. In addition, promastigotes treated with helenalin acetate were subject to an ultrastructural and biochemical investigation. The lethal action of the compound was investigated by fluorescence-activated cell sorting and related techniques to detect alterations in reactive oxygen species (ROS) content, plasma membrane permeability, and mitochondrial membrane potential. Helenalin acetate significantly reduced the mitochondrial membrane potential and the mitochondrial structural damage was also confirmed by transmission electron microscopy, displaying an intense organelle swelling. No alteration of plasma membrane permeability or ROS content could be detected. Additionally, helenalin acetate significantly increased the production of nitric oxide in peritoneal macrophages, probably potentiating the activity against the intracellular amastigotes. Helenalin acetate could hence be a useful anti-leishmanial scaffold for further optimization studies.

## 1. Introduction

Leishmaniases are listed as neglected tropical diseases by the World Health Organization [1]. Nowadays, these diseases affect 98 countries. In Brazil, an increased number of cases has been observed in recent years, accompanied by a geographical spread [1]. Worldwide, an estimated 1.3 million new cases and 20,000–30,000 deaths are reported annually; around 310 million people are at risk of an infection [1].

Depending on the parasite species and determinants of susceptibility of the vertebrate hosts, *Leishmania* infections present different clinical forms: cutaneous, mucocutaneous, and visceral leishmaniasis (VL) [2,3]. Untreated VL accounts for the majority of deaths reported for the leishmaniases [1]. In VL, the parasites affect mainly the spleen, liver, and bone marrow, where they multiply inside macrophages, causing organ hypertrophy (hepatosplenomegaly) and dysfunction [4]. Besides the high prevalence, impact, and risk among the world population, existing treatments for VL exhibit strong limitations, including high toxicity leading to severe adverse effects, high costs, parasite resistance, and hospitalization due to parenteral administration [5,6]. Therefore, new therapies for this disease are desirable.

Natural products traditionally play an important role in search for new therapeutics. Their large structural diversity of secondary metabolites is a source of novel chemical structures as starting points for drug development [7,8,9,10,11]. Natural sesquiterpene lactones (STLs) are widespread secondary metabolites in plants of the family Asteraceae [12]. This class of compounds are known for a wide range of biological activities such as anticancer [13,14], gastric cytoprotective [15] but also cytotoxic [16] effects, and in vitro anti-trypanosomatid activities [17,18,19,20,21,22,23].

**Figure 1:**
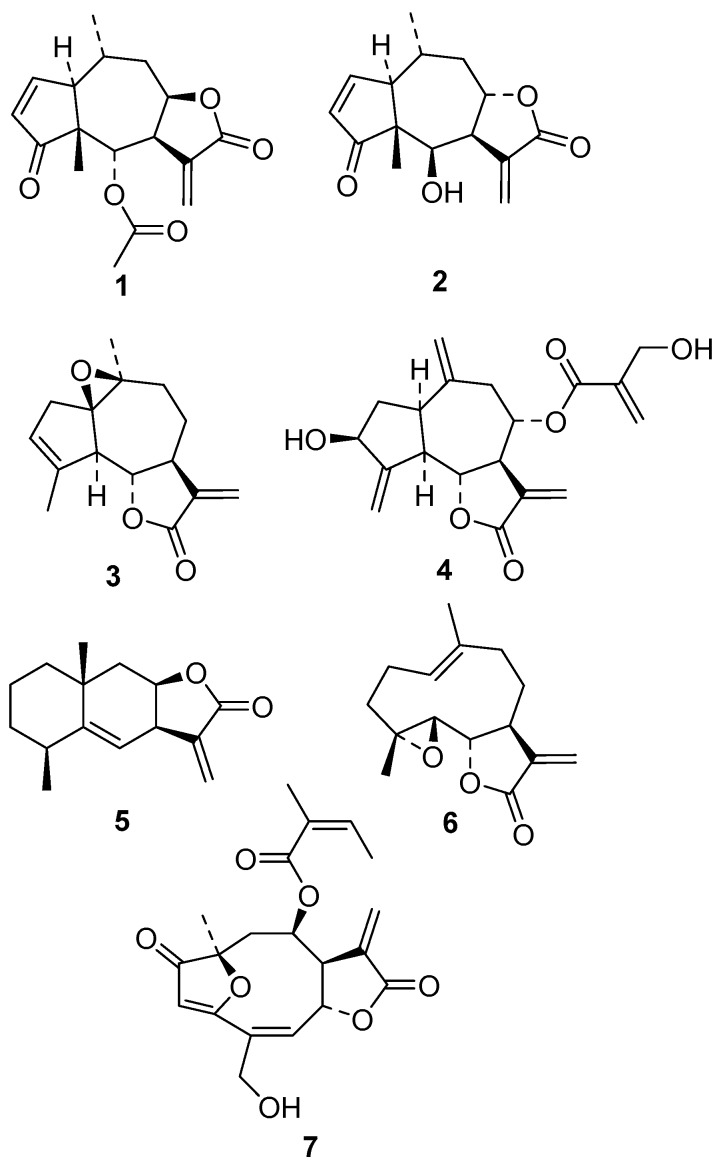
Structures of the tested sesquiterpene lactones (STLs). (**1**) Helenalin acetate; (**2**) Mexicanin I; (**3**) Arglabin; (**4**) Cynaropicrin; (**5**) Alantolactone; (**6**) Parthenolide; (**7**) Budlein A.

In this work, we evaluated the in vitro activity of the pseudoguaianolides mexicanin I and helenalin acetate; the guaianolides arglabin and cynaropicrin; eudesmanolide alantolactone; germacranolide parthenolide; and furanoheliangolide budlein A (Figure 1) against promastigotes and intracellular amastigotes of *Leishmania* (Leishmania) *infantum*, the etiologic agent of VL in wide parts of South America and the Mediterranean region. Using different techniques, we studied the mechanism of action of the most active and selective STL in *L.* (L.) *infantum*, evaluating different parameters of the parasite such as plasma membrane permeability, reactive oxygen species (ROS) content, and mitochondrial membrane potential (Δψ_m_) as well as the production of nitric oxide (NO) by host cells.

## 2. Results and Discussion

### Anti*-L.* (L.) *infantum* Activity of STL

Seven representative STLs (Figure 1) were chosen for this study since they had demonstrated different levels of in vitro anti-trypanosomatid activity against *Trypanosoma brucei rhodesiense* and *Leishmania donovani* (axenic amastigotes) in previous studies [22,23]. Their activity against *L.* (L.) *infantum* as well as their cytotoxicity and selectivity is demonstrated in Table 1. All compounds displayed in vitro activity against *L.* (L.) *infantum* promastigotes, with IC_50_ values in a concentration range between 3 and 59 μM. The pseudoguaianolides helenalin acetate (**1**) and mexicanin I (**2**) showed the most potent activity against the intracellular amastigotes and highest selectivity indices (S.I. = IC_50_(cytotox.)/IC_50_(amastigote)), with IC_50_ values of 1.15 μM (S.I. 7) and 1.73 μM (S.I. 5.3), respectively. Argablin (**3**) and cynaropicrin (**4**) also demonstrated selectivity against the intracellular amastigotes, but were about 6-fold less effective than the compounds **1** and **2**, demonstrating IC_50_ values of 7.33 (S.I. 5.3) and 6.88 μM (S.I. 4.8), respectively. Compounds **1** and **2** were about 15-fold more effective than miltefosine, the standard drug used to treat VL. Effects of **1** on intracellular amastigotes as observed by light microscopy are shown in Figure S1, Supplementary Material.

The unesterified parent compound of **1**, helenalin, and **2** were previously shown to have activity against *L.* (L.) *mexicana* at low concentrations, one of the species responsible for cutaneous leishmaniasis [20]. Considering the selectivity index and the high potency against promastigotes and intracellular amastigotes, compound **1** was selected for further studies aiming at biochemical and ultrastructural investigations in *L.* (L.) *infantum* parasites after treatment.

**Figure 2:**
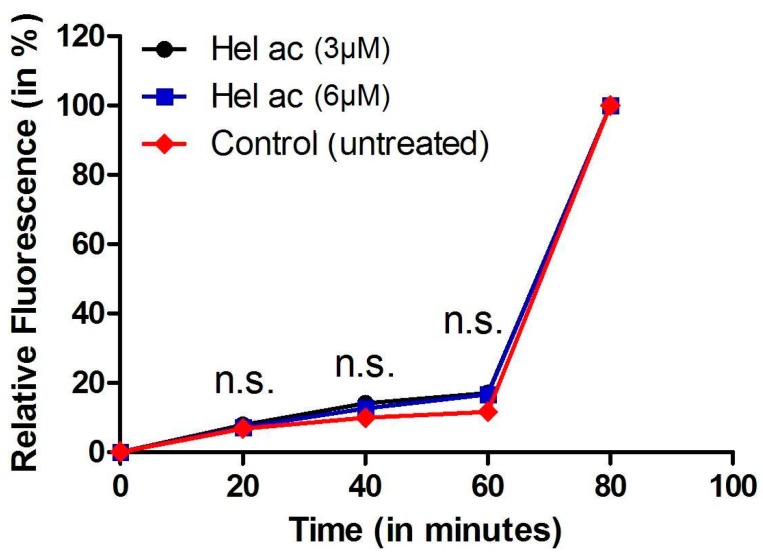
Permeability of *L.* (L.) *infantum* plasma membrane incubated with compound **1** assessed spectrofluorimetrically by the fluorescence of the SYTOX Green. Promastigotes were treated with **1** (3 and 6 μM) for 60 min. At the end of this treatment, Triton X-100 was used for 100% permeabilization. Dimethyl sulfoxide (DMSO) was used as internal control and did not cause any alteration in membrane permeability (data not shown).

The parasite’s plasma membrane regulates the transport of nutrients, pH homeostasis, and homeostasis of other ions [24]. Thus, we investigated the effect of **1** on the parasite plasma membrane using the probe SYTOX Green. This probe shows fluorescence when it gets in contact with nucleic acids, reaching the intracellular milieu via a severely damaged plasma membrane [25]. No significant increase of SYTOX Green fluorescence could be detected after treatment with compound **1** (3 and 6 μM) during 60 min (Figure 2), in comparison with untreated parasites. Thus, it can be concluded that **1** has no influence on the plasma membrane permeability of the parasite.

**Figure 3:**
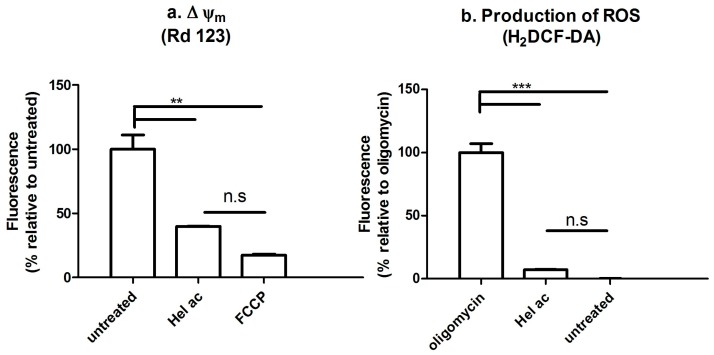
(**a**) Depolarization of the mitochondrial membrane potential (Δψ_m_) and (**b**) production of reactive oxygen species (ROS) of *L.* (L.) *infantum* promastigotes treated with compound **1** for 1 h. (**a**) Promastigotes were treated with **1** (6 μM) and then stained with rhodamine 123 (0.3 μg/mL) or (**b**) H_2_DCFDA (5 μM). Data were normalized to the intensity of Rd123 fluorescence achieved in non-depolarized untreated promastigotes (**a**) or H_2_DCFDA fluorescence achieved in promastigotes treated with oligomycin (**b**). In both cases, fluorescence was measured in 10,000 cells using a FL1 at 530 ± 15 nm on a Flow Cytometer Attune Nxt. Error bars depict the mean and standard deviation (SD) of duplicate samples. For each assay, one representative of two independently performed experiments is shown. n.s, not significant; *** indicates significant differences with the control at *p* < 0.001; ** indicates *p* < 0.01.

It is known that vital processes of *Leishmania* promastigotes are irreversibly affected by helenalin at short periods of exposure (3 h) [20]. Having ruled out the plasma membrane as target, the next step was the investigation of a possible influence of helenalin acetate (**1**) on the mitochondria of *L.* (L.) *infantum* promastigotes, a recognized target for a number of antileishmanial compounds [26]. *Leishmania* is known to have a single mitochondrion as major supplier of cellular energy—i.e., ATP—through the metabolism of the respiratory chain [27]. The mitochondrial membrane potential (Δψ_m_) is a crucial component in ATP production and its maintenance is essential for the parasite survival. Therefore, disturbances in the Δψ_m_ of *L.* (L.) *infantum* promastigotes treated with **1** were investigated using the fluorescent probe rhodamine 123. Rhodamine 123 (Rd123) is a fluorescent cationic dye that accumulates into polarized mitochondria. Depolarization, i.e., a decrease in Δψ_m_, results in a lower accumulation of rhodamine and, consequently, in a reduced fluorescence [28]. Parasites treated for 1 h with compound **1** at a concentration representing the previously determined IC_99_ value (6 μM), decreased the fluorescence of Rd123 by 60% in comparison to untreated parasites (Figure 3a). This effect on the Δψ_m_ was similar to that obtained with 10 μM carbonyl cyanide 4-(trifluoromethoxy) phenylhydrazone (FCCP), a known mitochondrial uncoupler. A previous study with parthenolide (**6**) against axenic amastigotes (extracellular) of *L.* (L.) *amazonensis*, also demonstrated a mitochondrial depolarization with this sesquiterpene lactone [21]. The exact mechanism by which **1** or **6** affect the Δψ_m_ of *Leishmania* remains to be elucidated.

Derangements in Δψ_m_ are usually linked to overproduction of ROS, resulting in mitochondrial dysfunction and ultimately cell death [29,30]. Accordingly, the production of ROS in promastigotes treated with compound **1** was determined using the dye 2′,7′-dichlorodihydrofluorescein diacetate (H_2_DCFDA) and flow cytometry. The fluorescence intensity of this dye has been assumed to be proportional to the redox state of the parasite. In contrast to oligomycin (20 μM), a mitochondrial respiratory chain inhibitor of the ATP synthase (complex V), which increased the ROS production over time, promastigotes treated with **1** showed no alteration of ROS after 1 h of treatment (Figure 3b).

The impairment in Δψ_m_ accompanied by ROS generation is commonly described for STLs in cancer cell lines [31,32]. It was suggested that, in these cells, STLs cause a knockdown of crucial enzymes that participate in the redox defense metabolism, resulting in ROS accumulation [33]. Similarly, some STLs have been described to induce ROS generation concomitant with reduction of glutathione (GSH) content in *Leishmania* promastigotes. GSH is an important metabolite of the redox defense metabolism of the parasite as a precursor for the synthesis of trypanothione. It was proposed that STLs react with sulfhydryl groups by the Michael-type addition; thus, impairment of redox defense metabolism of parasites should not be ruled out in *Leishmania* [34]. However, the absence of an increase in ROS generation in promastigotes treated with **1**, even in disturbed mitochondria as indicated by the affected Δψ_m_, suggests that the redox defense metabolism was probably not affected by this STL throughout the incubation period. Likewise, psilostachyin C—another STL—also demonstrated a leishmanicidal effect through a lethal mechanism other than oxidative stress or reduction of GSH content in the parasite [34]. In summary, the mitochondrial dysfunction provoked by STLs in *Leishmania* may have distinct mechanisms.

**Figure 4:**
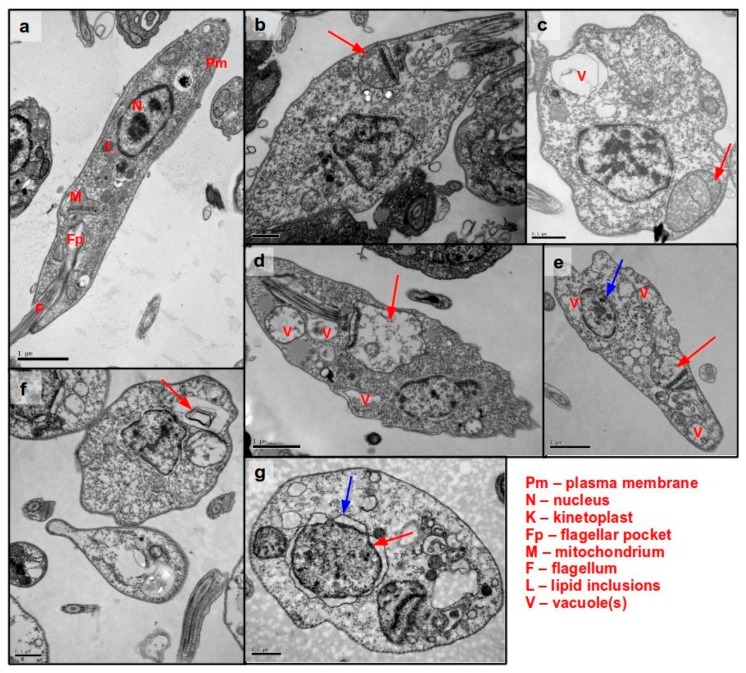
Transmission electron microscopy imaging of *L.* (L.) *infantum* promastigotes treated with helenalin acetate (**1**). 2 × 10^7^ promastigotes/well were incubated with **1** for different periods of time. (**a**) Untreated control. (**b**) 0.5 h; (**c**) 1 h; (**d**) 2 h; (**e**,**f**) 3 h; (**g**) 4 h. The figure shows representative images taken from one out of two independent experiment. The observed ultrastructural observations appeared consistently in both independent Transmission Electron Microscopy (TEM) experiments.

In addition to these biochemical investigations, an ultrastructural study using Transmission Electron Microscopy (TEM) was used to investigate the alterations in *L.* (L.) *infantum* promastigotes treated with compound **1** at 1 μM over different incubation times. **1** induced early morphological and ultrastructural alterations in promastigotes after 30 min of incubation, with increasing damage throughout the time interval studied, 4 h (Figure 4). The integrity of plasma membrane and pericellular microtubules was preserved over 4 h, corroborating the aforementioned SYTOX Green assay (Figure 4). Similarly, **1** induced no alteration of the plasma membrane of *L.* (L.) *mexicana* promastigotes after a long-term treatment [20]. The previously observed mitochondrial dysfunction could now be confirmed by a visible swelling of the organelle (Figure 4b–f, red arrows) during the treatment with **1**. The strong vacuolization of the cytoplasm was associated with autophagic processes as judged by presence of multivesicular structures (Figure 4f). Autophagy can be triggered by the cell for energy production, cell recycling, or death signalling [35]. Detachment of the nuclear membrane (Figure 4e, blue arrow) was initially observed after 3 h followed by chromatin decondensation after 4 h of incubation (Figure 4g, red arrow). Usually, this event precedes the DNA fragmentation which has also been reported to occur in *L.* (L.) *mexicana* promastigotes after long treatment with unesterified helenalin [20]. In summary, the ultrastructural analysis showed that compound **1** induced a rounded amastigote-like morphology (compare Figure 4a with Figure 4g) with intense cytoplasmatic vacuolization, formation of autophagosomes, swelling of the mitochondrion, and chromatin decondensation (Figure 4g).

**Figure 5:**
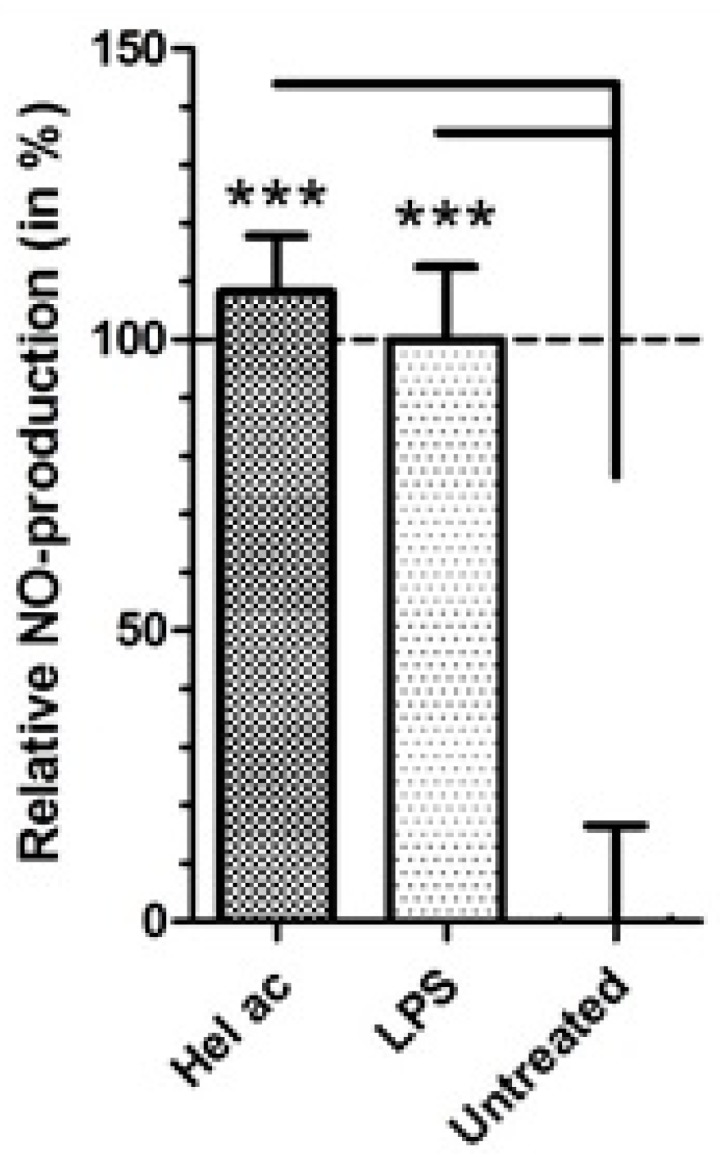
Production of nitric oxide (NO) by peritoneal macrophages of BALB/c mice treated with helenalin acetate (**1**). The production of NO was determined colorimetrically by the Griess assay. The data were blanked and normalized to the value of control cells (untreated macrophages); 1 × 10^5^ macrophages/well were incubated with (**1**) at 3 μM for 24 h. Untreated = Untreated macrophages; lipopolysaccharide (LPS) = 5 μg/mL lipopolysaccharide (positive control); Hel ac = helenalin acetate (**1**). Error bars depict the mean and SD of duplicate samples. *** α = 0.001. One representative of two independently performed experiments is shown.

Besides direct effects on the parasite, antileishmanial compounds can also trigger a lethal effect via macrophage activation mechanisms. Macrophages play an important role in the vertebrate immune system and represent the primary cells infected by *Leishmania*. Their activation profile is decisive for the resolution of the infection [36,37]. It has been demonstrated that macrophage-mediated killing of *Leishmania* could be an event associated with the up-regulation of NO. After stimulation with lipopolysaccharide (LPS), macrophages are activated to produce NO from arginine, controlling the proliferation of intracellular parasites such as *L.* (L.) *major* [38]. Considering the higher activity of **1** against the intracellular amastigotes of *L.* (L.) *infantum* as compared to extracellular promastigotes (Table 1), we investigated the possibility of the host cell participating in the elimination of the parasites. The NO levels of peritoneal macrophages treated with **1** at 3 μM were determined after 24 h incubation using the colorimetric Griess assay [39]. Our data demonstrated that **1** significantly increased the production of NO at similar levels as cells treated with LPS (positive control) (Figure 5). This result suggests that **1** elicited an immunomodulatory effect that may have contributed to reduce the infection by *L.* (L.) *infantum* via an NO-mediated mechanism.

## 3. Materials and Methods

### 3.1. Compounds

The STLs investigated in this study were previously isolated in our laboratories (T.J.S., F.B.C.) from plants of the family Asteraceae. Helenalin acetate (**1**) and mexicanin I (**2**) were isolated from *A. montana* L. and *Arnica acaulis* (Walter) Britton, Sterns and Poggenb., respectively. The guaianolides arglabin (**3**) and cynaropicrin (**4**) originate from *Artemisia glabella* Kar. Et Kir. Fl. Alt. and *Cynara cardunculus* L., respectively. Alantolactone (**5**) is an eudesmanolide isolated from *Inula helenium* L. The germacranolide parthenolide (**6**) and the furanoheliangolide budlein A (**7**) were isolated from *Tanacetum parthenium* (L.) Sch. Bip. and *Aldama robusta* (Gardner) E.E.Schill and Panero (formerly: *Viguiera robusta*). Further details on the origin of these compounds and their purity were published previously [18,22,23]. In all cases, stock solutions were prepared with dimethyl sulfoxide (DMSO) which were subsequently diluted with the respective medium to the specific concentration used in the various bioassays.

### 3.2. Animals

Golden hamsters (*Mesocricetus auratus* Waterhouse) were used for the maintenance of the *L.* (L.) *infantum* (MHOM/BR/1972/LD) culture. Young female BALB/c mice (*Mus musculus* L.) with a weight of 18–24 g were used as a source of peritoneal macrophages.

Animals were provided by the animal facility of the Adolfo Lutz Institute, São Paulo, Brazil. The animals received water and food ad libitum and were kept in sterilized cages. The procedures involving animals were realized in agreement with the *Guidelines for the Care and Use of Laboratory Animals* from the *National Academy of Sciences*, USA. The project received ethical approval by the *Research Ethics Commission* of the *Adolfo Lutz Institute/ Pasteur Institute* (CEUA-IAL/Pasteur 02/2011), São Paulo, Brazil.

### 3.3. Leishmania (*Leishmania*) infantum Promastigote, Peritoneal Macrophages, and NCTC Cells Culture

*L.* (L.) *infantum* (MHOM/BR/1972/LD) promastigotes were maintained in M-199 medium supplemented with 10% heat-inactivated fetal bovine serum (FBS) (Gibco, Thermo Fisher Scientific, São Paulo, Brazil), 0.25% hemin (Sigma-Aldrich), and 5% human urine at 24 °C. Amastigotes were obtained from the spleen of previously infected hamsters by differential centrifugation. Macrophages were collected from the peritoneal cavity of BALB/c mice by washing with RPMI-1640 medium supplemented with 10% FBS, and were maintained at 37 °C in a 5% CO_2_-humidified incubator. Murine fibroblasts NCTC (clone L929 ATCC) were used to evaluate the cytotoxicity of the tested compounds. NCTC cells were maintained in M-199 medium supplemented with 10% FBS and 20 μg/mL gentamicin at 37 °C in a 5% CO_2_-humidified incubator.

### 3.4. Determination of 50% Inhibitory Concentration (IC_50_) against Leishmania and 50% Cytotoxicity Concentration (CC_50_) against NCTC

#### 3.4.1. Promastigotes

Compounds were dissolved in DMSO and diluted in M-199 medium in 96-well microplates, with the highest concentration of 150 μM. Promastigotes of *L.* (L.) *infantum* in the late growth-phase were seeded at 1 × 10^6^/well and incubated with the compounds for 48 h. The viability of cells was determined using the MTT assay [40]. An internal control group was used with 0.5% DMSO (maximal concentration). Miltefosine was used as standard drug.

#### 3.4.2. Intracellular Amastigotes of *L.* (L.) *infantum*

Murine peritoneal macrophages were collected from the peritoneal cavity of BALB/c mice, and the macrophages were seeded at 1 × 10^5^/well for 24 h in a 16-well slide. Amastigotes were prepared (as described previously), seeded at a ratio 1:10 (macrophages:amastigotes) and kept at 37 °C in a 5% CO_2_-humidified incubator for 24 h. Test compounds were incubated to the highest concentration of 100 μM with infected macrophages for 120 h. Miltefosine was used as a standard drug. Subsequently, the cells were fixed with methanol, stained with Giemsa (Merck KGaA, Darmstadt, Germany), and observed using a light microscope. The parasite burden was determined by the number of infected macrophages out of 400 cells.

#### 3.4.3. Cytotoxicity

NCTC cells were seeded at 6 × 10^4^ in 96-well microplates and incubated with the compounds for 48 h. An internal control group was used with 0.5% DMSO (maximal concentration). The quantification of viable cells was assessed by the MTT assay [40]. The selectivity index (SI) was determined using the following equation: SI = CC_50_ in NCTC cells / IC_50_ against amastigotes.

### 3.5. Ultrastructural Analysis of Cellular Damage in Promastigotes Treated with Helenalin Acetate, Using TEM

Ultrastructural studies were performed with promastigotes treated with compound **1** by TEM. *L*. (L.) *infantum* promastigotes in the late growth phase were washed and incubated with 120 μM of **1** (corresponding to the IC_99_ value obtained with 1 × 10^6^ parasites/well) at 2 × 10^7^/well in 200 μL M-199 medium. Compound incubation was performed for 0.5, 1, 2, 3, and 4 h under the previously described growth conditions. After centrifugation at 2800 rpm for 10 min, the parasites were fixed with 1 mL glutaraldehyde and the sample processing was performed according to Duarte et al. [41]. The TEM imaging was performed on a JEOL 1011 transmission electron microscope (Peabody, MA, USA).

### 3.6. Determination of Plasma Membrane Permeability in Helenalin Acetate-Treated Promastigotes

Late-growth-phase (non-stationary) promastigotes were incubated with the probe SYTOX Green (Molecular Probes Inc., Eugene, OR, USA), as previously described [42]. Concentrations of **1** were chosen in accordance with the respective IC_50_ and IC_99_ values. **1** was added at 3 and 6 μM to 1 × 10^6^ cells/well, at 24 °C. The fluorescence was measured every 20 min (0–80 min). At the end of the assay (80 min), 0.1% Triton X-100 was added to all wells to obtain the maximum permeabilization of parasites. Fluorescence intensity was determined using a fluorimetric microplate reader (FilterMax F5 Multi-Mode Microplate Reader-Molecular Devices, Sunnyvale, CA, USA) with excitation and emission wavelengths of 485 and 520 nm, respectively. The following internal controls were used during the evaluation: (i) the background fluorescence of the compound at the respective wavelengths; (ii) the possible interference of DMSO; (iii) untreated promastigotes; and (iv) medium without any cells. Samples were tested in duplicate.

### 3.7. Determination of the Mitochondrial Membrane Potential (Δψm) and ROS by Fluorescence-Activated Cell Sorting

The Δψ_m_ and ROS content of promastigotes treated with compound **1** were determined using the fluorescence probes rhodamine 123 and H_2_DCFDA (Molecular Probes Inc., Eugene, OR, USA), respectively, and further evaluated by flow cytometry [25,43]. Briefly, 2 × 10^6^ promastigotes/well cultured until the late growth-phase were treated with 12 μM of **1** (corresponding to the IC_99_ value obtained with 1 × 10^6^ parasites/well). The parasites were incubated in HANKS’ balanced salts solution (HBSS; Sigma-Aldrich, Carlsbad, CA, USA), supplemented with 10 mM d-Glucose, at 26 °C for 1 h. Afterwards the parasites were washed and stained with rhodamine 123 (0.3 μg/mL) or H_2_DCFDA (5 μM) for 15 min under absence of light. The washed parasites were resuspended in PBS and the fluorescence was measured in a Flow Cytometer Attune Nxt (ThermoFisher Scientific, Waltham, MA, USA) using the forward scatter (FSC, relative cell size) and the side scatter detectors (SSC, cell granulometry or internal complexity), FL1-A (detecting fluorescence emission 530 ± 15 nm), and Attune Nxt software. As control, untreated promastigotes (not stained with Rd123 or H_2_DCFDA) served to monitor the basal fluorescence. FCCP (10 μM) and oligomycin (20 μM) provided information on the maximal mitochondrial depolarization and maximal ROS production, respectively. Samples were tested in duplicate. Two independent assays were performed.

### 3.8. Production of Nitric Oxide (NO) in Macrophages Treated with Helenalin Acetate

The NO production of macrophages treated with **1** was performed using the Griess assay [39]. Briefly, 1 × 10^5^/well peritoneal macrophages of BALB/c mice were seeded overnight in a 96-well plate. Fresh medium containing compound **1** at 3 µM (corresponding to the CC_25_ value obtained with NCTC cells) or 5 µg/mL LPS (positive control) were added and incubated for 24 h at 37 °C. Afterwards the NO content of each of the cultures’ supernatant was analysed using the Griess assay. The samples’ NO content was determined by means of a standard curve prepared with NaNO_2_ at concentrations from 0 to 400 μM.

### 3.9. Statistical Analysis

Data processing and statistical analyses was done with GraphPad Prism^®^ 5.0 (GraphPad Software, Inc. La Jolla, CA, USA). For determination of antileishmanial activity and NCTC cytotoxicity, the IC_50_ and CC_50_ values were calculated after normalization using sigmoidal dose-response curves. The evaluation of statistical significance of multiple pairs was done with a Tukey's range test, which corrects for the increased probability of a type I error during multiple comparison.

## 4. Conclusions

The present study revealed the in vitro anti-*Leishmania* (L.) *infantum* efficacy of seven STLs isolated from plants of the Asteraceae family. Helenalin acetate (**1**) and mexicanin I (**2**) showed the highest potency of all tested compounds and a certain selectivity against *L.* (L.) *infantum* intracellular amastigotes. Helenalin acetate (**1**) affected mitochondria at an early phase during incubation, causing depolarization of the mitochondrial membrane potential. These early events could be related to altered ATP production, since the mitochondrion is a pivotal organelle for bioenergetic homeostasis. Additionally, it was demonstrated that the activity of **1** against intracellular amastigotes could be, at least in part, related to an increased NO-production of the host cells since an elevated release of NO was induced by the compound in peritoneal macrophages. Helenalin acetate and, possibly, other STLs such as mexicanin I, may thus represent interesting scaffolds for the synthesis of derivatives with potent and selective activity against *L.* (L.) *infantum*.

## Figures and Tables

**Table 1:** In vitro antileishmanial activity and mammalian cytotoxicity of sesquiterpene lactones (STLs).

Compounds	*L.* (L.) *infantum* Promastigotes	*L.* (L.) *infantum* Amastigotes	Cytotoxicity	SI (NCTC/Amastigote)
	IC_50_ (μM) (SD)	IC_50_ (μM) (SD)	CC_50_ (μM) (SD)	
Helenalin acetate (**1**)	3.53 (0.2)	1.15 (0.22)	8.12 (1.47)	7.0
Mexicanin I (**2**)	4.89 (1.2)	1.73 (0.7)	9.20 (2.74)	5.3
Arglabin (**3**)	29.98 (2.4)	7.33 (4.0)	39.35 (6.38)	5.3
Cynaropicrin (**4**)	30.59 (0.61)	6.88 (3.5)	33.54 (7.39)	4.8
Alantolactone (**5**)	9.94 (0.14)	n.a.	11.04 (1.61)	-
Parthenolide (**6**)	59.13 (4.00)	89.20 (-)	58.18 (9.14)	<1
Budlein A (**7**)	28.00 (7.87)	n.a.	9.05 (1.86)	-
Miltefosine	16.69 (3.49)	17.80 (1.39)	116.70 (5.30)	6.5

IC_50_: 50% inhibitory concentration; CC_50_: 50% cytotoxic concentration; n.a.: not active to the highest concentration of 100 μM; SI: selectivity index based on CC_50_ in murine fibroblasts (NCTC)/IC_50_ in amastigotes.

## Supplementary Materials

Supplementary materials are available online. Figure S1: Helenalin acetate activity against intracellular *L.* (L.) *infantum* amastigotes (GIEMSA-stained light microscopy images).
