# Supplementary material for: Investigation of the Anti-Leishmania (Leishmania) infantum Activity of Some Natural Sesquiterpene Lactones
Source: Molecules. 2017 Apr 25;22(5):685. doi: 10.3390/molecules22050685 (PMC6154613; doi:10.3390/molecules22050685)
Supplement: Supplementary file 1 [file molecules-22-00685-s001.pdf]

Supplementary Material

Investigation of the Anti-*Leishmania* (Leishmania) *infantum* Activity of Some Natural Sesquiterpene Lactones

Imke F. Wulsten [1†], Thais A. Costa-Silva [2], Juliana T. Mesquita [2], Marta L. Lima [2,3], Mariana K. Galuppo [2], Noemi N. Taniwaki [2], Samanta E. T. Borborema [2], Fernando B. Da Costa[4], Thomas J. Schmidt [5,*], and Andre G. Tempone [2,*]

Figure S1: Helenalin acetate activity against intracellular *L.* (L.) *infantum* amastigotes

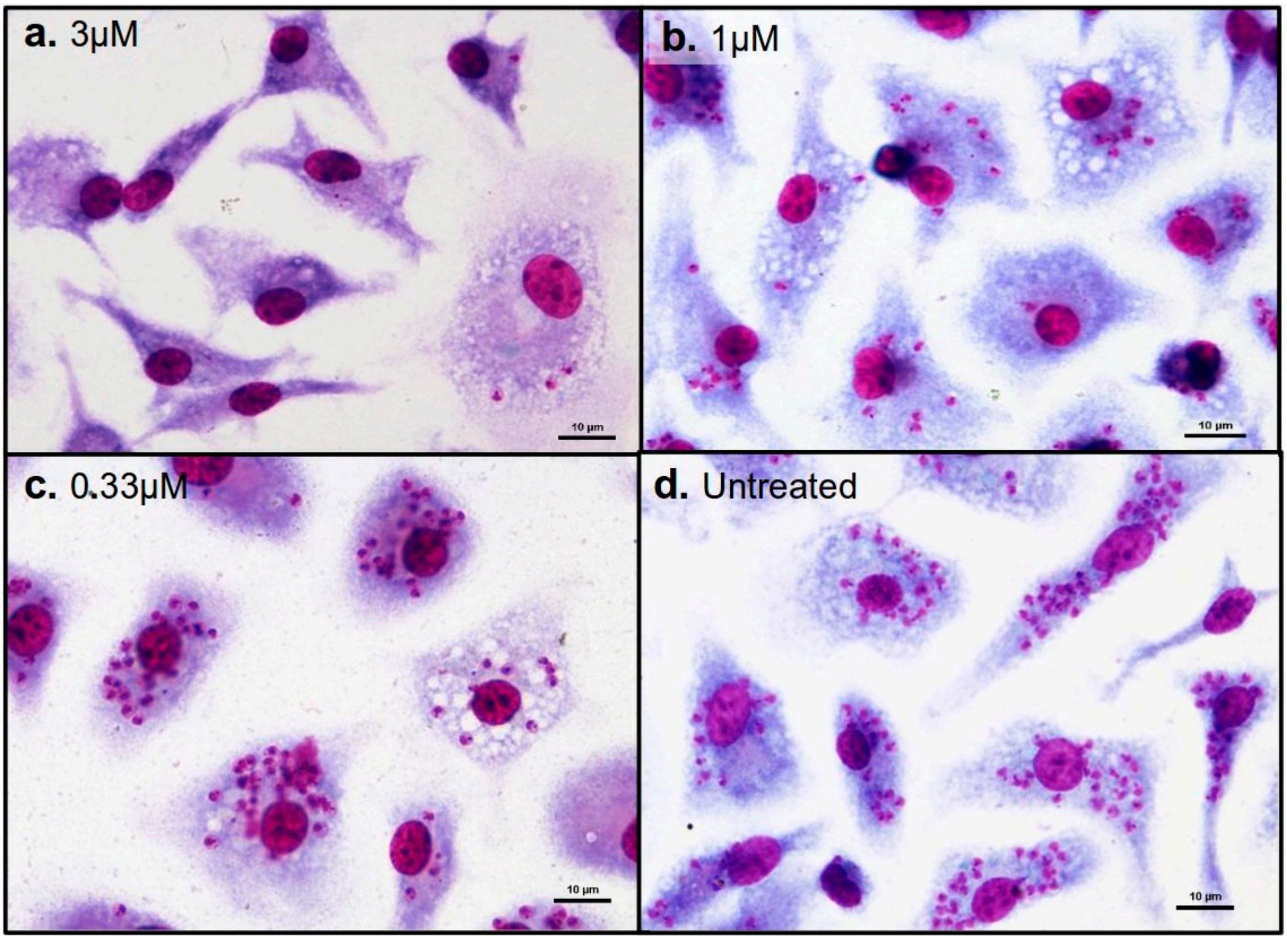

Helenalin acetate activity against intracellular amastigotes. GIEMSA-stained light microscopy slides of amastigote-infected macrophages which were treated with different concentrations of helenalin acetate. **(a)** 3 µM. The image depicts very few infected macrophages with each very few amastigotes. Small and medium size vacuoles usually indicate that amastigote infection and resolution has taken place within the macrophages. Cells are well spread and display a normal morphology. Dividing macrophages were observed regularly at the highest tested compound concentration, comparable to the control. **(b)** 1 µM. A large number of infected macrophages is visible, but these contain few to a medium number of amastigotes. Vacuoles of various sizes display resolved amastigote infections. **(c)** 0.33 µM. The image shows an infection rate with a relatively high amastigote number. **(d)** Untreated, but infected macrophages. The control image depicts a high macrophage infection rate with a high amastigote number. (Source: own research. Representative images are chosen.).
